# Supplementary material for: See-N-Seq: RNA sequencing of target single cells identified by microscopy via micropatterning of hydrogel porosity
Source: Commun Biol. 2022 Jul 30;5:768. doi: 10.1038/s42003-022-03703-3 (PMC9338959; doi:10.1038/s42003-022-03703-3)
Supplement: Supplementary file 2 — Supplementary Information [file 42003_2022_3703_MOESM2_ESM.pdf]

Supplemental Information for

See-N-Seq: RNA Sequencing of Target Single Cells Identified by Microscopy via Micropatterning of Hydrogel Porosity

Authors: Jeong Hyun Lee[1,2], Emily S. Park[1,2], Jane Ru Choi[1,2], Kerryn Matthews[1,2], Alice V. Lam[1,2], Xiaoyan Deng[1,2], Simon P. Duffy[1,2,3], and Hongshen Ma[1,2,4,5]

Affiliations
[1]Department of Mechanical Engineering, University of British Columbia
[2]Centre for Blood Research, University of British Columbia
[3]British Columbia Institute of Technology
[4]School of Biomedical Engineering, University of British Columbia
[5]Vancouver Prostate Centre, Vancouver General Hospital

Table of Contents

Fig. S1. Detailed molecular workflow for sequencing library preparation.

Fig. S2. Flow sorting of Jurkat-Raji immune synapse doublets.

Fig. S3. Volcano plots of comparisons between bulk samples and between single cell samples.

Fig. S4. Scatter and correlation between single cells for single Raji-Jurkat synapse at 24 hours.

Table S1. List of genes in heatmap

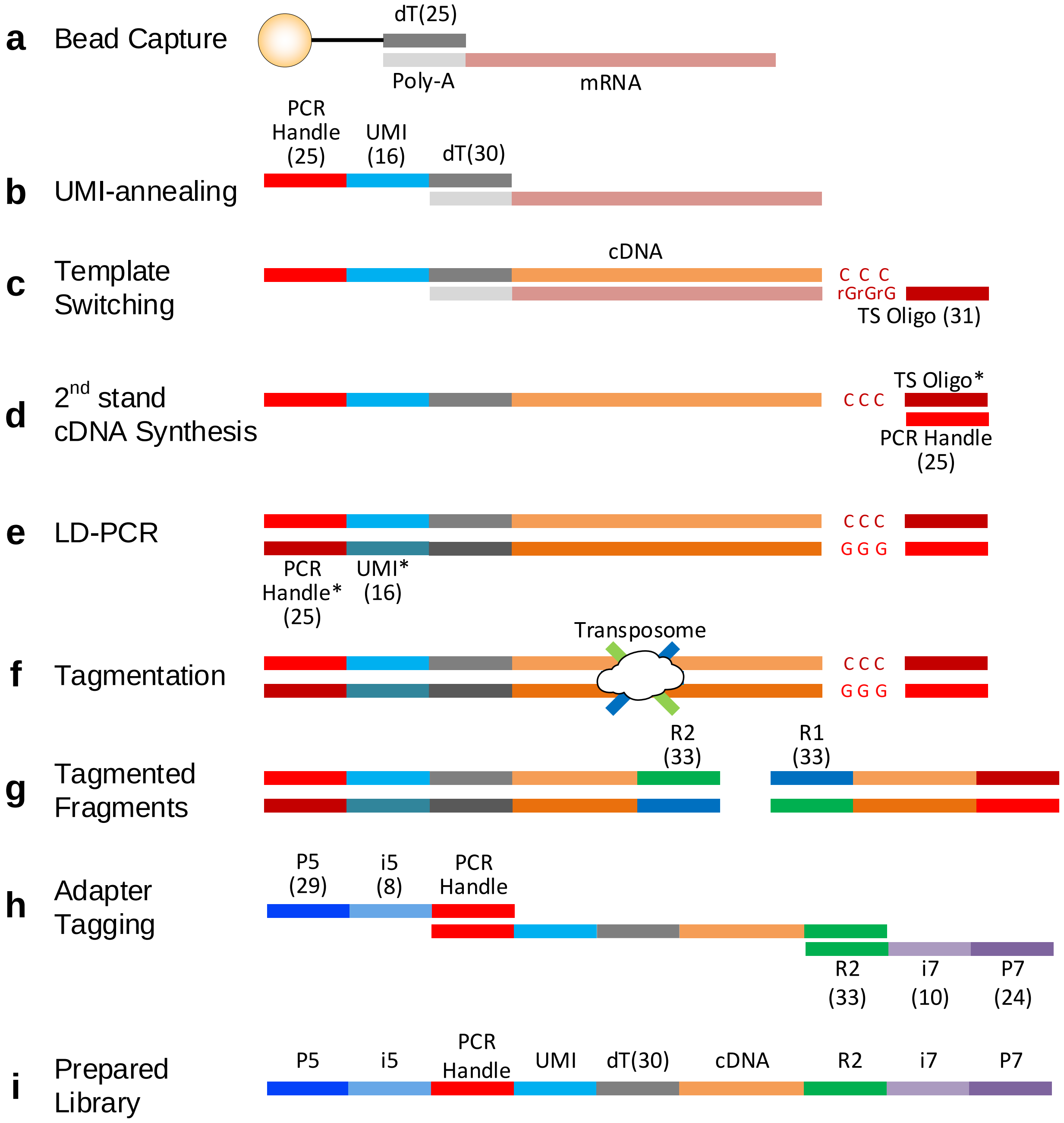

Fig. S1. Related to Figure 1; Detailed molecular workflow for sequencing library preparation. (**a**) Extracted mRNA is captured using oligo-dT beads. (**b**) The mRNA is eluted from the beads and annealed to oligonucleotides consisting of PCR handle, unique molecular index (UMI), and oligo-dT. (**c**) Reverse transcription is performed with a template switching oligo, which provides a PCR handle. (**d-e**) The reverse transcribed cDNA is then amplified by long distance PCR (LD-PCR). (**f-g**) The amplified cDNA is tagmented using the Illumina Nextera DNA flex kit. (**h**) After tagmentation with read sequence insertion, the sequencing adapters P7 and P5 are attached by PCR. (**i**) In order to specifically sequence the UMI on READ1, a custom primer is used, which contains P5, Illumina's i5 index and PCR handle. The prepared library is pooled to 6 µM and sequenced using Illumina Next-seq 550.

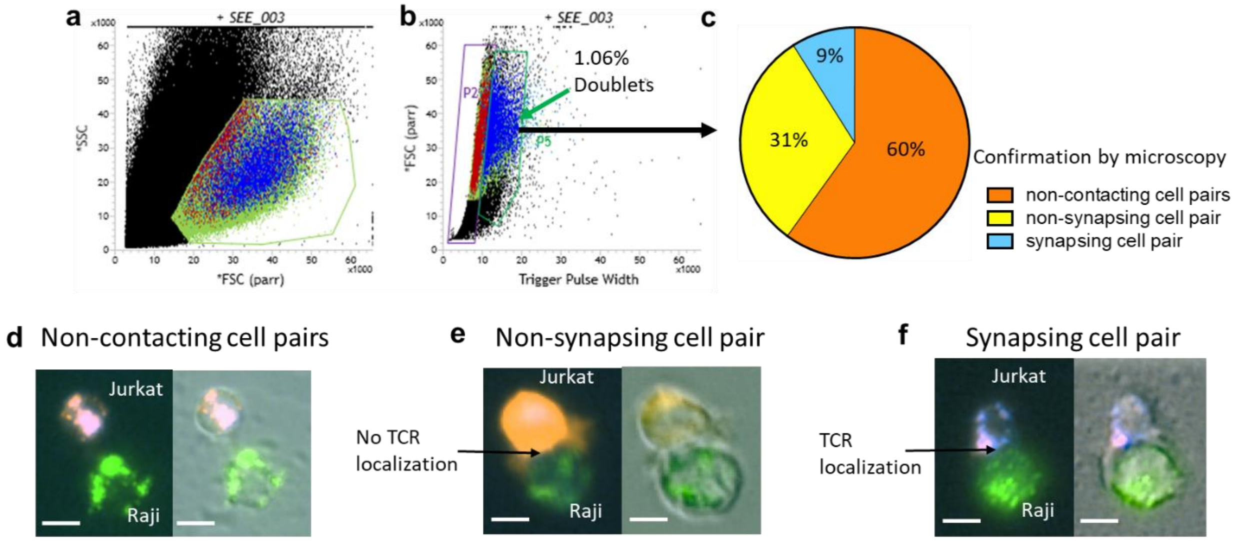

Fig. S2. Related to Figure 3; Flow sorting of Jurkat-Raji immune synapse doublets. (**a**) Forward Scatter (FSC) and Side Scatter (SSC) plot of viable Jurkat and Raji cells, (**b**) Gating for Doublet cells based on FSC and Trigger pulse width, showed only 1.06% of cells were doublets. (**c**) Doublets were sorted and were plated in imaging well plates for confirmation by microscopy. After counting of cells, 60% of doublets were shown to be (**d**) cell pairs or single cells not in contact with each other, 31% of cell doublets were (**e**) non-synapsing cell pairs showing no TCR localization by the absence of TCR staining, while 9% of cell doublets were (**f**) synapsing cell pairs as confirmed by the presence of TCR staining (blue). All scale bar = 10 µm.

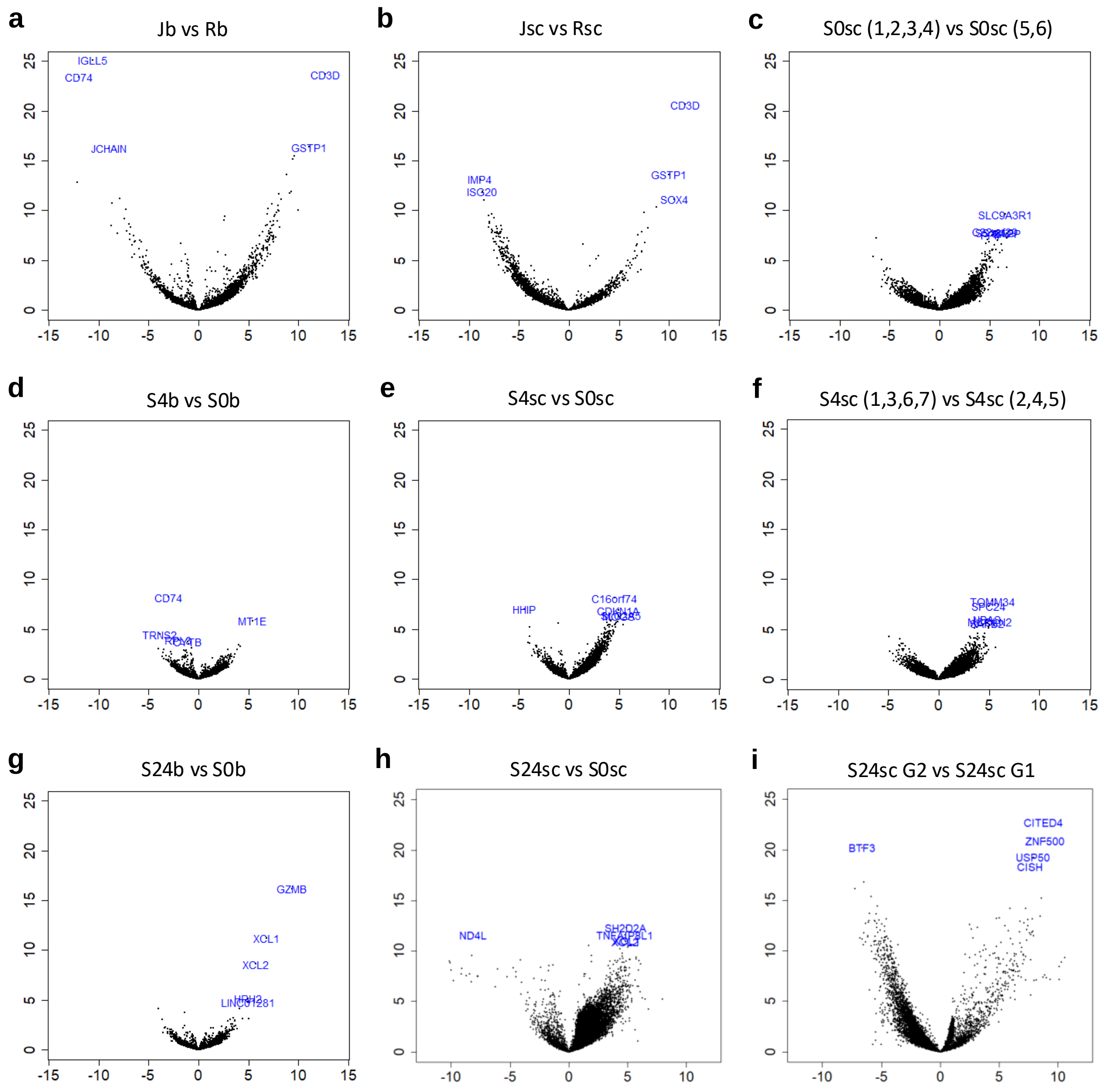

Fig. S3. Related to Figure 4; Volcano plots of comparisons between bulk samples and between single cell samples. The volcano plot shows DGE analysis between sample groups and sample subgroups of (**a**) Jurkat bulk and Raji bulk, (**b**) Single Jurkat and single Raji, (**c**) S0sc (1,2,3,4) and S0sc (5,6), (**d**) S4 (synapse 4 hour) bulk and S0 (synapse 0 hour) bulk, (**e**) Single S4 and single S0, (**f**) S4sc (1,3,6,7) and S4sc (2,4,5), (**g**) S24 (synapse 24 hour) bulk and S0 bulk, (**h**) Single S24 and single S0 and (**i**) S24scG2 and S24scG1. X-axis indicates $\log_2$-fold change, Y-axis indicates $-\log_{10}$ P-value. Top 5 genes that have the lowest P-value highlighted as a gene name (blue).

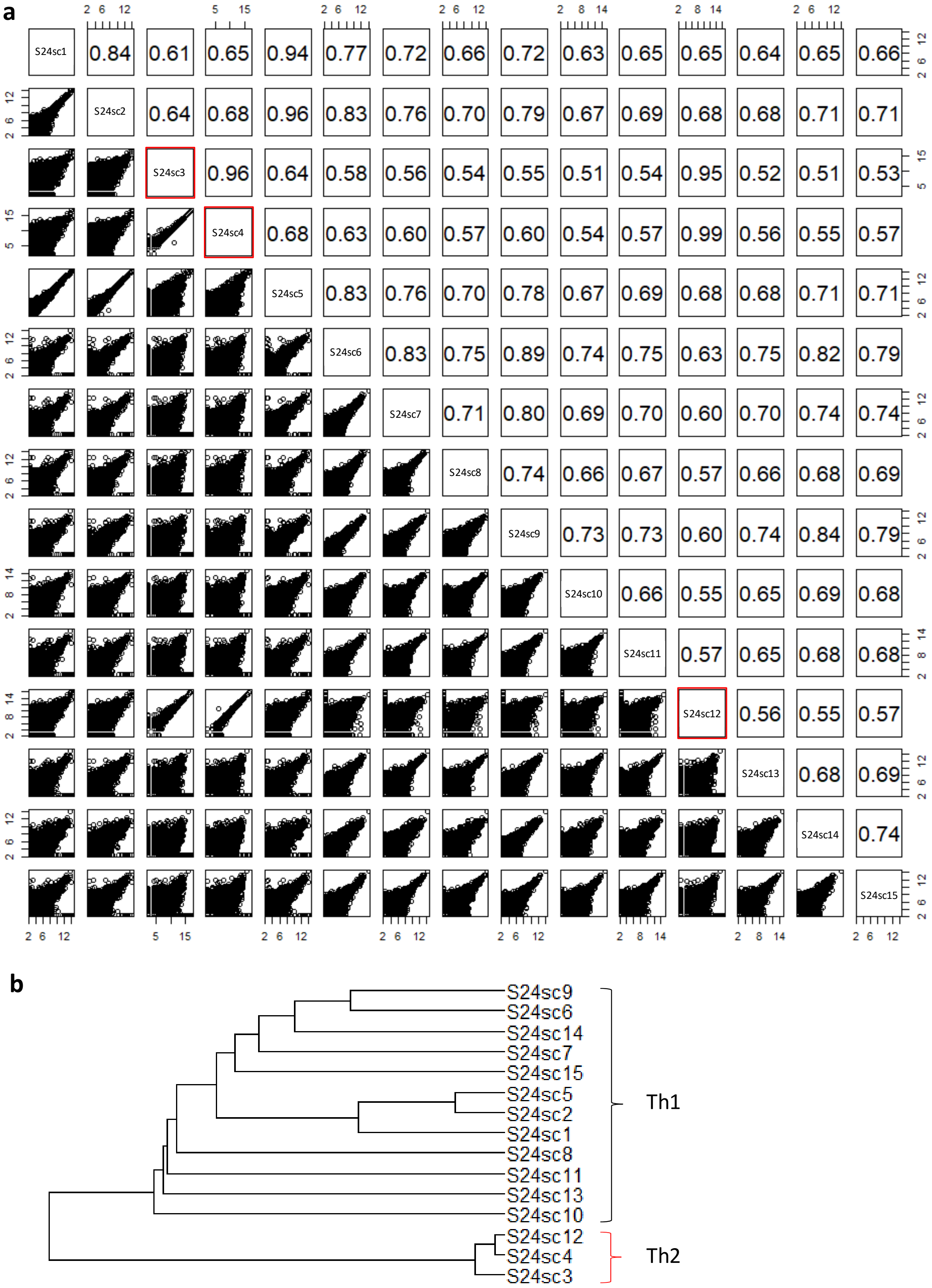
a
S24sc1 0.84 0.61 0.65 0.94 0.77 0.72 0.66 0.72 0.63 0.65 0.65 0.64 0.65 0.66
S24sc2 0.64 0.68 0.96 0.83 0.76 0.70 0.79 0.67 0.69 0.68 0.68 0.71 0.71
S24sc3 0.96 0.64 0.58 0.56 0.54 0.55 0.51 0.54 0.95 0.52 0.51 0.53
S24sc4 0.68 0.63 0.60 0.57 0.60 0.54 0.57 0.99 0.56 0.55 0.57
S24sc5 0.83 0.76 0.70 0.78 0.67 0.69 0.68 0.68 0.71 0.71
S24sc6 0.83 0.75 0.89 0.74 0.75 0.63 0.75 0.82 0.79
S24sc7 0.71 0.80 0.69 0.70 0.60 0.70 0.74 0.74
S24sc8 0.74 0.66 0.67 0.57 0.66 0.68 0.69
S24sc9 0.73 0.73 0.60 0.74 0.84 0.79
S24sc10 0.66 0.55 0.65 0.69 0.68
S24sc11 0.57 0.65 0.68 0.68
S24sc12 0.56 0.55 0.57
S24sc13 0.68 0.69
S24sc14 0.74
S24sc15
b
S24sc9
S24sc6
S24sc14
S24sc7
S24sc15
S24sc5
S24sc2
S24sc1
S24sc8
S24sc11
S24sc13
S24sc10
S24sc12
S24sc4
S24sc3
Th1
Th2

Fig. S4. Related to Figure 4; Correlation between single cells for single Jurkat-Raji synapse at 24 hours. (a) Scatter and correlation plot. The Log_2-CPM of each sample was plotted in a matrix of scatter plots. The scatter plot in the lower-left matrix position shows the relationship between compared samples. Each dot represents one gene and each axis indicates Log_2-CPM values, while the number in the reflected upper-right matrix position indicates the Pearson correlation coefficient for compared samples. Sample group 2 for S24sc are highlighted in red. **(b) Phylogenetic tree.** The root node divided into two separated groups, consist of S24scG1 (Th1-like) and S24scG2 (Th2-like).

Table S1. Related to Fig. 4; List of genes in heatmap

Specificity	Genes
Th1 related genes	*TMEM205, AP1S3, GTF2H3, REL, DNAJC19, GATAD1, SREBF2, NSMF, SIL1, TBC1D10C, METTL7A, PIDD1, TSPAN17, GIHCG, DCPS, DOK2, GOLGA8B, TOM1, SNX2, L3MBTL2, YJU2, KDM2B, PRKD3, CYB561A3, PLCG1, THUMPD3-AS1, TAP1, UACA, EML2, TTPAL, BACH2, KCNMB4, CKAP4, IKZF2, AFF3, HERC3, DTX3L, MED15, MTRR, SLC39A10, HINFP, ABHD5, CD22, PI4KA, PARP10, STAMBP, TESK1, CTSB, ADA2, APOBEC3G, TRAF3IP2, MSL1, IFT81, TINF2, VNN2, LAIR1, GALE, MKRN1, TBL1X, FILIP1L, PLGRKT, ICK, GBP2, PSMB8-AS1, PECAM1, PIGO, ZADH2, DDX20, DEXI, TMEM106A, SLAMF1, S100PBP, FURIN, KDM3B, MTRNR2L2, GPR18, ABCC1, ARHGAP9, SLX4, ATG9B, CXCR5, SNTA1, GNB5, SOCS4, SSH1, ANXA2R, CLDND1, ZNF302, MORN4, APOL1, DBP, EHD4, SLC30A6, SENP1, GCH1, PARP9, HDAC10, TESPA1, ZDHHC7, USP18*
Th2 related genes	*PRKCQ, IL2RA, RXRA, FTL, RPS15A, TPT1, SLC25A1, TPM3, NEDD8, TCHP, SPIB, ATP5PF, C1orf53, COX6A1, SLIRP, HADHB, HNRNPUL1, TXNDC9, CCL22, LSM10, DFFA, ZFAS1, CD164, PARD6B, CD27, MRPL55, ECH1, IFI30, LYRM9, ATP5F1A, MYL12A, VIM, ATP6V0E1, VCP, LRRFIP1, MEX3A, TNFRSF11A, NDUFB6, NOP56, HADHA, GTPBP4, HSPBP1, ZMAT3, MVD, SURF1, HMGA1, NARF, TMEM120B, EMG1, NOP10, SENP2, TAX1BP1, ANKRD33B, P2RX5, UBE2F, TYMP, HDAC2, SLC4A4, RYBP, TRAF1, WAC-AS1, ELMSAN1, TMBIM6, SYF2, GNB1, GLRX, FBXW7, MRPL20, MAP2K2, IDS, HPCAL1, FKBP3, PNP, MAP4K1, C1orf109, TULP3, WDR36, VIRMA, CHP1, FAM120A, CCT8, PGP, NSDHL, SQSTM1, RAB8A, SELENOW, IFIT5, ANXA11, ITGB1BP1, NOP16, SEPTIN11, HARS, FDFT1, NELFB, BRSK2, ISL2, PRMT6, GIMAP1, TRAT1, COL6A1*
