# Supplementary material for: See-N-Seq: RNA sequencing of target single cells identified by microscopy via micropatterning of hydrogel porosity
Source: Commun Biol. 2022 Jul 30;5:768. doi: 10.1038/s42003-022-03703-3 (PMC9338959; doi:10.1038/s42003-022-03703-3)
Supplement: Supplementary file 3 — Description of Additional Supplementary Files [file 42003_2022_3703_MOESM3_ESM.pdf]

Description of Additional Supplementary Files

File name: Supplementary Data 1
Description: Source data for graphs in the paper
