# Supplementary material for: See-N-Seq: RNA sequencing of target single cells identified by microscopy via micropatterning of hydrogel porosity
Source: Commun Biol. 2022 Jul 30;5:768. doi: 10.1038/s42003-022-03703-3 (PMC9338959; doi:10.1038/s42003-022-03703-3)
Supplement: Supplementary file 5 — Reporting Summary [file 42003_2022_3703_MOESM5_ESM.pdf]

nature portfolio

Corresponding author(s): Hongshen Ma
Last updated by author(s): Oct 5, 2021

Reporting Summary

Nature Portfolio wishes to improve the reproducibility of the work that we publish. This form provides structure for consistency and transparency in reporting. For further information on Nature Portfolio policies, see our Editorial Policies and the Editorial Policy Checklist.

Statistics

For all statistical analyses, confirm that the following items are present in the figure legend, table legend, main text, or Methods section.

n/a	Confirmed	
☐	☒	The exact sample size (*n*) for each experimental group/condition, given as a discrete number and unit of measurement
☐	☒	A statement on whether measurements were taken from distinct samples or whether the same sample was measured repeatedly
☐	☒	The statistical test(s) used AND whether they are one- or two-sided<br>*Only common tests should be described solely by name; describe more complex techniques in the Methods section.*
☒	☐	A description of all covariates tested
☒	☐	A description of any assumptions or corrections, such as tests of normality and adjustment for multiple comparisons
☐	☒	A full description of the statistical parameters including central tendency (e.g. means) or other basic estimates (e.g. regression coefficient) AND variation (e.g. standard deviation) or associated estimates of uncertainty (e.g. confidence intervals)
☐	☒	For null hypothesis testing, the test statistic (e.g. *F*, *t*, *r*) with confidence intervals, effect sizes, degrees of freedom and *P* value noted<br>*Give P values as exact values whenever suitable.*
☒	☐	For Bayesian analysis, information on the choice of priors and Markov chain Monte Carlo settings
☐	☒	For hierarchical and complex designs, identification of the appropriate level for tests and full reporting of outcomes
☐	☒	Estimates of effect sizes (e.g. Cohen's *d*, Pearson's *r*), indicating how they were calculated

Our web collection on statistics for biologists contains articles on many of the points above.

Software and code

Policy information about availability of computer code

Data collection	NIS-Elements AR(version 5.21.03), UMI_tools (version 1.0.0), Cutadapt (version 2.4), STAR aligner (2.7.2b), Subread (2.0.0), samtools (version 1.8)
Data analysis	R (version 3.6.1) in R studio (version 1.2.1335) with 'edgeR' package, version 3.26.8

For manuscripts utilizing custom algorithms or software that are central to the research but not yet described in published literature, software must be made available to editors and reviewers. We strongly encourage code deposition in a community repository (e.g. GitHub). See the Nature Portfolio guidelines for submitting code & software for further information.

Data

Policy information about availability of data

All manuscripts must include a data availability statement. This statement should provide the following information, where applicable:
- Accession codes, unique identifiers, or web links for publicly available datasets
- A description of any restrictions on data availability
- For clinical datasets or third party data, please ensure that the statement adheres to our policy

Data available NCBI BioProject ID PRJNA615050 (https://dataview.ncbi.nlm.nih.gov/object/PRJNA615050?reviewer=rfsh6sfnfbj1raegr7trionf5v)

Field-specific reporting

Please select the one below that is the best fit for your research. If you are not sure, read the appropriate sections before making your selection.

☒ Life sciences ☐ Behavioural & social sciences ☐ Ecological, evolutionary & environmental sciences

For a reference copy of the document with all sections, see nature.com/documents/nr-reporting-summary-flat.pdf

Life sciences study design

All studies must disclose on these points even when the disclosure is negative.

Sample size	No sample size was calculated.
Data exclusions	Amplified cDNA from single cell samples were tested using qPCR. Samples with Ct values >37 for CD3 and >30 for GAPDH were excluded from sequencing. The frequency of qPCR failure was 15.7%. Samples with sequencing reads <10,000 were excluded from analysis. The frequency of sequencing failure was 2.5%. (SI Methods page 4)
Replication	RNA sequencing data on Jurkat-Raji immunological synapses represent biological replicates because library preparation was performed on different cells across different preparations.
Randomization	Randomization was not done as the study was comparing Jurkat-Raji cells forming an immune synapse vs. Jurkat-Raji cells not forming an immune synapse. All cells came from the same biological source.
Blinding	Blinding was not relevant to our study as we had to use imaging to visualize cells in an immune synapse in order to select for them.

Reporting for specific materials, systems and methods

We require information from authors about some types of materials, experimental systems and methods used in many studies. Here, indicate whether each material, system or method listed is relevant to your study. If you are not sure if a list item applies to your research, read the appropriate section before selecting a response.

Materials & experimental systems

n/a	Involved in the study	
☐	☒	Antibodies
☐	☒	Eukaryotic cell lines
☒	☐	Palaeontology and archaeology
☒	☐	Animals and other organisms
☒	☐	Human research participants
☒	☐	Clinical data
☒	☐	Dual use research of concern

Methods

n/a	Involved in the study	
☒	☐	ChIP-seq
☒	☐	Flow cytometry
☒	☐	MRI-based neuroimaging

Antibodies

Antibodies used	BV421 Mouse Anti-Human CD3, Clone UCHT1, Cat # 562426, BD Bioscience
Validation	Product detail available at manufacturer's website.

Eukaryotic cell lines

Policy information about cell lines

Cell line source(s)	Cells are purchased from ATCC or were received as a gift
Authentication	Jurkat cells were authenticated by the presence of CD3 staining and CD3 qPCR.<br>Raji cells were authenticated by the presence of CD19 qPCR.<br>UC13-EGFP and UC13-mCherry were authenticated by the presence of green and red fluorescence as well as the presence of EGFP and mCherry genes by qPCR.
Mycoplasma contamination	Cell lines were not tested for mycoplasma contramination.
Commonly misidentified lines (See ICLAC register)	*Name any commonly misidentified cell lines used in the study and provide a rationale for their use.*
